# News and Notes

**Published:** 1902-05

**Authors:** 


### NEWS AND NOTES.

(We are indebted to the *Medical Age* for many of these notes.)

---

THE *Atlanta Medical and Surgical Journal* and *Southern Medical Record* having been consolidated into the ATLANTA JOURNAL-RECORD OF MEDICINE, exchanges will please be sent only to the latter. We are getting many duplicates.

---

THE plague mortality in India is still on the increase.

---

AN epidemic of diphtheria is reported among the Pueblo Indians.

---

THE Congress of French Physicians of America will be held in June at Quebec.

---

THE University of Paris has 245 professors; of these, 76 are in the medical faculty.

---

CHOLERA has broken out among the pilgrims at Medina. Medina is 250 miles north of Mecca, Arabia.

---

REPORTS from Alaska show a prevalence of scurvy in many cities on account of the lack of fresh vegetables.

---

THE sculptor Harro Magnussen has been commissioned to execute a marble bust of Haeckel, professor of zoölogy and comparative anatomy at Jena.

---

DR. HERMAN KNAPP, the celebrated ophthalmologist of the College of Physicians and Surgeons of New York, recently celebrated his seventieth birthday.

THE Cross of the Legion of Honor has been conferred by President Loubet, of France, on William H. Tolman, of New York, for his work in behalf of the poor of that city.

---

SMALLPOX in the United States, as officially reported from December 28, 1901, to March 7, 1902, amounted to 20,044 cases with 615 deaths. The total for the corresponding period in 1901 was 7,637 cases and 104 deaths.

---

THE medical department of the University of Pennsylvania will establish a postgraduate medical school this spring to correspond to the "Ferien-Curse" of the German universities. The courses will be from four to six weeks' duration.

---

THE thirty-first German Congress of Surgery was held at Berlin April 2 to 5. Seven communications were read upon the treatment of wounds; Gussenbauer, Petersen and von Mikulicz discussed cancer, and several others spoke upon the subject of abdominal surgery.

---

FIRST ANNUAL MEETING OF THE GEORGIA STATE SOCIOLOGICAL SOCIETY.—The object of the Society is to unite all the professional, business and industrial interests of the State in the most efficient manner possible; to foster and encourage the study and investigation of all social questions that pertain to the welfare of mankind. Its special object is to ascertain the primal cause of crime, vice and disease, instituting measures of prevention by eliminating the cause of each. Also to disseminate such knowledge as will tend to uplift and better the condition of the human race.

Permanent committees as follows have been established and chairmen appointed for three years to study and investigate the subjects assigned to them:

1. Crime and its Prevention, including a Study of the Criminal and his Development, Dr. S. G. C. Pinkney, Atlanta, Ga.

2. Insanity, including Mental Degeneracy, Dr. T. O. Powell, Milledgeville, Ga.

3. Alcoholism, Dr. W. B. Parkes, Atlanta, Ga.

4. Tuberculosis, Dr. J. N. Brawner, Atlanta, Ga.

5. Diseases and Sanitary Science, Dr. J. Lawton Hiers, Savannah, Ga.

6. Education, Public Schools and Child Labor, Prof. B. M. Zettler, Atlanta, Ga.

7. The Negro Problem—Its Relation to the White Race, Rev. C. B. Wilmer, Atlanta, Ga.

8. Publication, Lectures and Distribution of Literature.

9. Legislation and Uniform Laws. (The last two are composed of the chairmen of the other committees.)

10. Auditing, Rev. L. M. Davis, Atlanta, Ga.

The Society will hold annual meetings in different sections of the State, at which time the committees will make reports of work accomplished. Papers and addresses on social questions will be presented and discussed by members of the Society. By investigation, discussion, education, lectures and legislation the Society hopes to aid in the prevention and control of crime, vice and disease.

Men and women are eligible to membership on same conditions.

Annual dues, one dollar. Dues before next meeting will be received as dues for the year 1902.

The next meeting will be held in Atlanta, Ga., Tuesday, Wednesday and Thursday, June 24th, 25th and 26th, 1902.

The prospects are encouraging for an interesting, profitable meeting.

Several papers on sociological questions have been promised besides the committee reports which will add materially to the interest of the meeting. The medical profession is cordially invited to attend the meeting, join the association and take part in the discussions.

Any one desiring to join may send their name, occupation and address with one dollar dues to Dr. J. N. Brawner, Secretary, 401-2 Prudential building, Atlanta, Ga.

---

COMMENCEMENT EXERCISES OF THE ATLANTA COLLEGE OF PHYSICIANS AND SURGEONS, APRIL 5, 1902.—One of the largest classes in the history of the Atlanta College of Physicians and Surgeons was graduated last night at the Grand opera house in the presence of a splendid gathering which filled the house from pit to balcony.

The exercises were unusually interesting and consisted of reports from the Dean, Dr. W. S. Kendrick, of the Medical Department, and from the Dean of the Pharmaceutical Department, the conferring of degrees by Judge Howard Van Epps, the delivery of the annual address by Rev. Theron Rice, and incidental music.

Out of a class of 120 Dr. L. L. Moye received first honor; Dr. C. D. Jeffries, second honor, and Dr. J. M. Barnett, third honor. The following received honorable mention: Dr. M. M. Head, Dr. R. B. Ridley, Dr. L. C. Rouse, Dr. A. Conter, Dr. A. W. Ralls and Dr. O. E. Kupferman.

The three doctors appointed to the staff of the Grady Hospital were William Arthur Selman, O. T. White and W. A. Jeffries.

The Pharmaceutical class, numbering 131 graduates, coming from eleven States, made a splendid showing. Under the direction of Dr. George F. Payne, Dean, this department of the College has made wonderful progress, increasing from 30 to 131 graduates. A telegram of congratulation wrs received by Dr. Payne from William A. Allison, editor of *The Druggists' Circular*, one of the leading publications of the kind in the country.

Following is a list of those who received diplomas in the Medical Department:

Section 1.—Howard Emerson Ezell, Albert Cronin Ives, Oscar E. Kupferman, W. Walker Evans, Hiram Griffith Williams, Thomas Walter Dorsett, Clarence Landers Avers, John Mervin McKenzie, Herbert Clifton Mallory, Oliver R. Fore, Augustus Conter, Adiel Roscoe Scott, Theodore Maddox, Eugene M. Herbert, Thomas Eugene Hewitt, George Henry Harper, Oliver Thomas White, Henry Jackson Goodwyn, Theo. Dorsett, James Maxey Dell, Percy Ellis Godbold and William Deitz.

Section 2.—Luke G. Garrett, Webb Conn, Harry Edgar Rosser, Albert Leroy Crittenden, Joseph M. Boring, Baron Frances Cornett, Willard T. Bayles, Horace Ollie Sprrks, Charles Haggard, Hal Mabry Terry, Howard Clifton Derrick, George Capron Merriam, William Arthur Selman, Robert Berry Bledsoe, James Luther Hearn, Mather Marvin McCord, Clinton H. Ramsey, McLain Rogers, Joseph Frances Jones, Lamar Preston Fordham, John Harrison Hendrix and Landon Cheek Pitchford.

Section 3.—Albert Clarence Corry, Trigg Dean Larkin, Edward Colfax McCurdy, George Pinckney Callan, George Wallace Patterson, Willis Walley, John Brady Jackson, Edward A. McClellan, James Miller Barnett, T. E. McBride, Marion Gordon Hendrix, Wallace Leslie Britt, Charles Abercrombie Wilkins, Jasper Lee Jackson, Henry Harris Shipp, Maishall Martin Hearn, Whitman Holton, John Rodolph Jordan, Thomas Newton Berry, Joseph Colquitt Logan, Linton Stephen's Smith and Ror Blosser.

Section 4.—Clymer Defoor Jeffries, Joseph Hamilton Pankey, James Robert Hervey, Edward Leroy McIntosh, Louis Calvin Rouse, Claude E. Reitzel, Frank Daniel, Leon L. Moye, Reynold Price Baird, John Caldwell Calhoun, Homer Hodges Brinson, Jesse James Davis, Robert Henry Cochran, Emit Luther McCafferty, Alva Clay Jones, Eugene Clower, James Augustus Dorsett, Robert Abner Harris, Samuel Block McMillan, Daniel Porter Mixon, David Houston Chilton and J. Carlton Williams.

Section 5.—Robert Berrien Ridley, Jr., Samuel Tinson Shepherd, Henderson E. Watts, Oscar Tucker, Jesse Hill Campbell,

George Washington Baker, Mark Williams Hollinshead, Arthur William Rall, James Marcus Hughes, Marvin Monroe Head, Jesse Mark Hill, John Elbert Lester, Homer S. Stallings, Elden Harkness Foster, Michael Lawrence Cronin, Curtis Monroe Jackson, James Drew Ingram, James Ragan McEachern, Thomas Sargent Bailey, James Edward Allgood, William David Sellers and J. C. Thorn.

The report of the Dean showed the College to be in splendid condition. Following is the report:

MR. PRESIDENT AND GENTLEMEN: I have the honor to report that we present for graduation this evening the largest class of medical students since the adoption of the three-year system, and the largest class of pharmaceutical students in the whole history of the College.

This is the fourth commencement since the consolidation of the Atlanta and Southern Medical Colleges under the name of the present institution. Whatever doubt may have at first existed as to the wisdom of that movement has been dispelled by the present prosperous condition of the Atlanta College of Physicians and Surgeons. We are supported by the good will of the profession at large, and are making every effort, consistent with the most favorable surroundings, to maintain our prestige as the foremost medical institution in this section of the South. To this end, after the present commencement, graduates of this College will have attended four instead of three courses of lectures previous to the reception of the degree. The facilities we offer for scientific research and for outdoor clinical work are probably not equaled south of Baltimore.

In attendance upon lectures in the three departments of Medicine, Pharmacy and Dentistry there have been this year 482 men. Two hundred and fifty-five of these have been in the Medical Department, and 111 ask at your hands the degree of doctor of medicine; 131 have attended the College of Pharmacy, and 48 are candidates for the degree of graduates in pharmacy; 96 have been enrolled in the Dental Department, and of these a number will, at a future date, ask for the degree of doctor of dental surgery.

Of the 255 medical students, 154 are from Georgia, 31 from Alabama, 16 from Florida, 13 from Mississippi, 12 from Texas, 10 from South Carolina, 6 from North Carolina, 4 from Louisiana, 3 from Virginia, 2 from New York, 1 from Tennessee, 1 from Missouri, 1 from Kentucky and 1 from Indian Territory.

These have been classified as follows: First year, 49; second year, 48; third year, 24; fourth year, 137; post graduates, 6.

The faculty takes great pleasure in commending to the public every young gentleman who shall receive his diploma this evening. They have had opportunities of no mean degree, and their future attainments will doubtless reflect credit upon their alma mater. Respectfully submitted.

W. S. KENDRICK, Dean of the Faculty.

---

THE shocking intelligence of the death in the Philippines of Dr. L. B. Grandy has been received. As yet no details are obtainable, so that a more extended notice is reserved for the next issue of THE JOURNAL-RECORD. Elsewhere in this number is a communication from Dr. Grandy. In the personal letter which accompanied it there was a promise of a continued article, one part of which has been received, but before the remainder could be completed, Death's finger touched him and he slept. We feel all the sorrow that belongs to the loss of an old and valued friend.

